# Thermal processing of pomegranate seed oils underscores their antioxidant stability and nutritional value: Comparison of pomegranate seed oil with sesame seed oil

**DOI:** 10.1002/fsn3.3918

**Published:** 2024-01-04

**Authors:** Javad Tavakoli, Afsaneh Ghorbani, Abdollah Hematian Sourki, Askar Ghani, Aniseh Zarei Jelyani, Przemysław Łukasz Kowalczewski, Aynura Aliyeva, Amin Mousavi Khaneghah

**Affiliations:** ^1^ Department of Food Science and Technology, Faculty of Agriculture Jahrom University Jahrom Fars Iran; ^2^ Department of Horticultural Science, Faculty of Agriculture Jahrom University Jahrom Fars Iran; ^3^ Department of Food and Drug Shiraz University of Medical Science Shiraz Iran; ^4^ Department of Food Technology of Plant Origin Poznań University of Life Sciences Poznań Poland; ^5^ Department of Technology of Chemistry Azerbaijan State Oil and Industry University Baku Azerbaijan; ^6^ Department of Fruit and Vegetable Product Technology Prof. Wacław Dąbrowski Institute of Agricultural and Food Biotechnology – State Research Institute Warsaw Poland

**Keywords:** antioxidant activity, oxidative stability, phenolic compounds, *Punica granatum*, regeneration, tocopherols

## Abstract

In the present study, the oxidative stability and antioxidant activity of seed oils were investigated in three Iranian pomegranate cultivars, Shirin Khafr, Torsh Sabz, and Rabab, along with the sesame (*Sesamum indicume* L. cv Dezful) seed oil. Punicic acid was the primary fatty acid in the pomegranate seed oils, with contents ranging from 75.5 to 80.9% (w/w). The tocopherol levels in pomegranate seed oils ranged from 1439 to 2053 mg/kg, whereas the phenolics ranged from 130 to 199.3 mg/kg, respectively. Comparatively, in the seed oil of sesame “Dezful,” these substances' contents were 1053 and 79 mg/kg, respectively. Contrary to common perception, the seed oil of the three pomegranate cultivars cultivated in Iran had high oxidative stability and antioxidative activity during the 32 h of thermal processing at 170°C. The oxidation stability assayed by peroxide value, p‐anisidine value, and TOTOX index revealed that the pomegranate seed oils had a much higher resistance to the oxidation process than the sesame oil. The content of tocopherols increased during thermal processing due to the regeneration phenomenon. Tocopherols are not always free and may form a matrix with themselves or other compounds. Changes in the antioxidant activity during the thermal processing assessed by DPPH free radical scavenging power and by the FRAP test were consistent with those for the antioxidants. Therefore, these oils can be added to other edible oils as a natural antioxidant to improve their oxidative stability.

## Graphical abstract

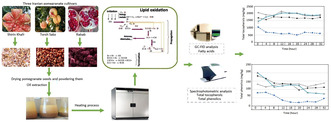

## 1 INTRODUCTION

Pomegranate (*Punica granatum* L.) belongs to a small plant family, Punicaceae. The origin of *P. granatum* is the Middle East, in particular the nowadays Iran, from which it later spread to other countries (Amri et al., 2017). Pomegranate fruit production placed fifth among the horticultural products in Iran and, with a production volume of more than 700,000 tons per year, amounts to a high share of the total fruit production in the country (Troujeni et al., 2018). Iranian Fars province is the highest pomegranate‐producing area in Iran, with an annual harvest of approximately 193,000 tons. The true berry fruit of pomegranate is consumed fresh or used in producing pomegranate juice and contains a high mass share of seeds that varies from 40 to 100 g/kg depending on the cultivar (Fadavi et al., 2006). Pomegranate seeds are post‐processing by‐products and contain substantial oils, proteins, sugars, and minerals (Tehranifar et al., 2010). In the pomegranate‐growing areas in Iran, the seeds are exclusively used for animal fodder.

Pomegranate seed oil makes up to 20% w/w of the seed and has high levels of nutritionally desirable unsaturated fatty acids. This oil contains one of the rare isomers of the conjugated linolenic acid called punicic acid, with documented health benefits (Carvalho et al., 2010; Özgül‐Yücel, 2005). The pomegranate seed oil also contains several antioxidant compounds at levels exceeding those of common edible oils: tocopherols, polyphenols, and sterols (Elfalleh et al., 2011; Fernandes et al., 2015; Gramza‐Michałowska et al., 2019; Habibnia et al., 2012; Juhaimi et al., 2017; Kmiecik et al., 2022). Given that more than 70% of the fatty acids in the pomegranate seed oil are punicic acid, which results in an overall high unsaturation of the oil, no strong oxidative stability is expected from that oil. Based on the above assumption, no extensive studies have been conducted on the oil oxidative stability at high temperatures and long exposure. Few studies have focused on the oxidative stability of pomegranate seed oil at temperatures below 100°C. The oxidative stability of that oil at 65°C showed that the combined use of pomegranate peel extract and butylated hydroxytoluene (BHT) antioxidant improved the oxidative stability of the pomegranate seed oil (Drinić et al., 2020).

The mechanisms of oxidative reactions and the action of antioxidant compounds at temperatures above 100°C differ from those at temperatures below 100°C (Amiri et al., 2021; Azizi et al., 2021; Choudhary et al., 2023; Gao et al., 2023; Mohamadi et al., 2023; Tayebeh et al., 2021). For example, increasing the content of tocopherol and polyphenolic compounds in edible oils is possible during thermal processing at 180°C for 20 h compared to their initial values (AlGhamdi et al., 2023; Veronezi & Jorge, 2018). Therefore, based on the initial properties of the edible oils, it is impossible to accurately estimate their oxidative stability at long‐time exposure to high temperatures (Tang et al., 2021; Ugarte‐Espinoza et al., 2021). For this reason, research on the oxidative stability and the antioxidative activity of pomegranate seed oils at temperatures above 100°C and under long exposure can provide good information about that oil. In order to know the heat resistance of pomegranate seed oil at high temperatures, seeds of three common varieties of pomegranate in Fars province (located in the southwest of Iran), namely Shirin Khafar, Torsh Sabz, and Rabab were collected, and their oil was extracted. Then, these mentioned oils' oxidative stability and antioxidant activity after 32 h of thermal processing at 170°C were investigated.

## 2 MATERIALS AND METHODS

### 2.1 Materials

The seeds of pomegranate cultivars Shirin Khafr, Torsh Sabz, and Rabab and the *Sesamum indicate* L. cv Dezful were prepared in Khafr city (in Fars province, Iran, autumn 2020) and kept at 4°C before oil extraction. Merck and Sigma‐Aldrich companies obtained the solvents and chemicals needed for this research. This research used sesame (*Sesamum indicate* L. cv Dezful) seed oil to compare with pomegranate seed oils. Among common edible oils, sesame seed oil has strong antioxidant activity. On the other hand, its unsaturation is also very high. For these reasons, this oil was used as a control.

### 2.2 Extraction of pomegranate seed oil

After drying in the shade, pomegranate and sesame seeds were powdered in a grinder and extracted with *n*‐hexane (1:4 w/v) by agitation for 24 h. After complete oil extraction, the hexane solvent was evaporated at 50°C using a vacuum evaporator (LaboxactSEM842, KNF, UK) (Asnaashari et al., 2015). The oil yield of seeds of pomegranate cultivars Shirin Khafr, Torsh Sabz, and Rabab was 16, 14, and 13%, respectively.

### 2.3 Heat processing

Samples of pomegranate seed oil (500 g) were poured into Erlenmeyer flasks and placed into a hot paraffin bath in an oven (170°C) for 32 h. After every 4 h, 20 g of oil sample was removed and stored at 4°C for further analysis (Tavakoli & Sorbi, 2017).

### 2.4 Fatty acid composition

The fatty acid composition of the vegetable oils was determined by gas–liquid chromatography according to the method previously established (Zarei Jelyani et al., 2021).

### 2.5 Cox value and iodin value

The method proposed by Tavakoli et al. (2016) was used to compute the calculated oxidizibility (Cox) value of the oils:

$$\mathrm{COX}\;\text{Value}=\frac{\left[\left(C18:1\;\left[\%\right]\right)+10.3\times \left(C18:2\;\left[\%\right]\right)+21.6\times \left(C18:3\;\left[\%\right]\right)\right]}{100}$$

The method described by Kmiecik et al. (2022) was applied to measure iodine value (IV). The calculation method is based on the percentage of hexadecenoic acid, octadecenoic acid, octadecadienoic acid, octadecatrienoic acid, eicosanoid acid, and docosenoic acid.

### 2.6 Acid value, peroxide value, p‐anisidine value, and TOTOX value

The determination of acid value, peroxide value, and *p*‐anisidine value (p‐AV) of the different oil samples was done based on the method ascribed by Tavakoli et al. (2016), Zarei Jelyani et al. (2021), and Asnaashari et al. (2015), respectively. The TOTOX value was determined based on the following formula:

TOTOX index = (2 × peroxide value) + *p*‐anisidine value.

### 2.7 Determination of DPPH radical scavenging activity and FRAP test

The DPPH radical scavenging assay and ferric‐reducing antioxidant power (FRAP) test of the studied oil were determined by the introduced method by Tavakoli et al. (2019).

### 2.8 Unsaponifiable matter content

The unsaponifiable matter content was determined by the method described by Tavakoli et al. (2016). For this purpose, the saponification of oil samples was first carried out using ethanolic KOH (1 N). Then, the extraction of unsaponifiable matter was carried out during two stages of washing with diethyl ether.

### 2.9 Total Phenolics and total tocopherol contents

The total phenolic content was measured according to the applied method by Mildner‐Szkudlarz et al. (2019) using high‐performance liquid chromatography (HPLC, Waters, Milford, MA) with an XBridge^TM^ C18 reversed‐phase column (4.6 × 100 mm; 3.5 μm) (Waters, Milford, MA).

The high‐performance liquid chromatography (HPLC) (WATERS, Alliance system, USA) technique was applied in order to determine the total tocopherol content, respectively (ISO9936, 2016).

### 2.10 Oxidative stability index

The oxidative stability index (OSI) was determined using a Rancimat model 743 (Metrohm, Herisau, Switzerland). For each test, 3 g of oil samples were analyzed. Temperature and airflow rate were set at 120°C and 15 L L/h, respectively (Tavakoli et al., 2016).

### 2.11 Statistical analysis

All experiments were done in three replications, and the results were analyzed using one‐way analysis of variance (ANOVA) implemented in MStat‐C v. 6.00. The Duncan's test implemented in MStat‐C was applied to assess the significance of differences among the mean values (α = 0.05) for each parameter, respectively.

## 3 RESULTS AND DISCUSSION

### 3.1 Chemical characteristics

#### 3.1.1 Fatty acid composition, cox value, and iodine value

Some characteristics of the seed oils from three pomegranate cultivars, Shirin Khafr, Torsh Sabz, and Rabab, and the sesame cultivar Dezful are presented in Table 1. The punicic acid was the predominant fatty acid of pomegranate seed oils and exceeded 75% oil but was not found in the sesame oil. Pomegranate seed oils contained a small amount of α‐linolenic acid (0.63 to 1.36%), less than the “Dezful” oil (4.60%). In contrast to the pomegranate seed oils, linoleic acid was predominant in the “Dezful” oil (48.1%). The linoleic acid content in pomegranate seed oils varied between 5.38 and 7.54%. The main saturated fatty acids in oils were palmitic acid and stearic acid, respectively. The highest amount of saturated fatty acids was observed in “Dezful” oil (13.56%) and the lowest in “Rabab” (5.23%), respectively. Among the double‐bonded fatty acids, the oleic acid content in pomegranate seed oils ranged between 5.97 and 8.36%, whereas in “Dezful” oil, it was 32%. Previous studies on pomegranate seed oil have identified punicic acid as the predominant fatty acid. The amount of this acid in the seed oil of 10 cultivars grown in Morocco was reported at 73.7 and 80.7% (Loukhmas et al., 2021). Also, in a study on the seed oil of six pomegranate cultivars cultivated in Georgia, the punicic acid content was determined at 78.3 and 83.4% (Pande & Akoh, 2009). The levels of punicic acid in the seed oil of six pomegranate cultivars in Turkey ranged between 71.2 and 77.6% (Juhaimi et al., 2017). However, the levels of punicic acid in the seed oil of the non‐edible cultivar Nana and the edible Tounsi from Tunisia were at 2.23 and 40.1%, respectively (Amri et al., 2017). The punicic acid content of the seeds of seven pomegranate cultivars grown in Spain ranged between 43.4 and 88.2% (Melgarejo & Artes, 2000). As such, the seed oils in the tested pomegranate cultivars from Iran contain punicic acid in the upper range from those reported to date. The fatty acid structure analysis showed that the pomegranate seed oils are rich in punicic acid, a conjugated linolenic acid (CLNA) component. These acids are rarely found in animal fats and are not commonly found in vegetable oils (Özgül‐Yücel, 2005). Studies have shown punicic acid has beneficial health effects such as antitumor activities, immune system regulators, anti‐atherosclerosis, and serum lipid lowering (Carvalho et al., 2010; Grossmann et al., 2010). Therefore, with high levels of punicic acid, the studied pomegranate seed oil may also have health‐benefiting properties.

**TABLE 1:** Chemical composition of the seed oils from three pomegranate cultivars (Shirin Khafr, Torsh Sabz, and Rabab) and one cultivar of sesame (Dezful) (unheated oils; initial values).

Parameters	Oil samples			
	“Shirin Khafr”	“Torsh Sabz”	“Rabab”	“Dezful”
Palmitic acid (16:0) (%)	3.02 ± 0.20 d	4.30 ± 0.17 b	3.23 ± 0.06 c	9.80 ± 0.06 a
Palmitoleic acid (16:1) (%)	Not detected	Not detected	Not detected	0.5 ± 0.04
Stearic acid (18:0) (%)	2.18 ± 0.16 c	2.43 ± 0.03 b	2.00 ± 0.10 c	3.60 ± 0.05 a
Oleic acid (18:1) (%)	6.37 ± 0.20 c	8.36 ± 0.34 b	5.97 ± 0.09 d	32.0 ± 1.3 a
Linoleic acid (18:2) (%)	5.38 ± 0.59 c	7.54 ± 0.04 b	5.99 ± 0.10 c	48.1 ± 1.6 a
α‐Linolenic acid (18:3) (%)	1.36 ± 0.03 b	0.63 ± 0.07 d	1.14 ± 0.05 c	4.60 ± 0.20 a
Punicic acid (18:3) (%)	80.9 ± 1.0 a	75.5 ± 1.3 b	80.0 ± 1.0 a	Not detected
Arachidic acid (20:0) (%)	0.43 ± 0.03 b	0.58 ± 0.03 a	Not detected	Not detected
Gondoic acid (20:1) (%)	Not detected	Not detected	0.69 ± 0.03 a	0.50 ± 0.10 b
Behenic acid (22:0) (%)	Not detected	Not detected	Not detected	0.48 ± 0.05
SFA (%)	5.62 ± 0.38 c	7.31 ± 0.21 b	5.23 ± 0.13 d	13.56 ± 0.32 a
MUFA (%)	6.37 ± 0.20 c	8.36 ± 0.34 b	6.66 ± 0.1 c	33.0 ± 1.3 a
PUFA (%)	87.7 ± 1.6 a	83.6 ± 1.3 b	87.1 ± 1.09 a	52.7 ± 1.46 d
USFA/SFA	16.75 ± 0.8 b	12.6 ± 0.2 c	17.93 ± 0.44 a	6.32 ± 0.2 d
MUFA/PUFA	0.07 ± 0.00 d	0.10 ± 0.00 b	0.08 ± 0.00 c	0.63 ± 0.01 a
PUFA/SFA	15.6 ± 0.77 b	11.45 ± 0.16 c	16.65 ± 0.43 a	3.9 ± 0.11 d
Iodine value (g *I* ^2^/100 g oil)	230 ± 4 a	219 ± 3 b	228 ± 3 a	123 ± 4 c
Calculated oxidizibility (Cox) value	18.4 ± 0.28 a	17.3 ± 0.3 b	18.2 ± 0.2 a	6.28 ± 0.15 c
Unsaponifiable matters content (% of oil)	2.83 ± 0.50 a	1.95 ± 0.10 d	2.25 ± 0.15 b	2.21 ± 0.04 c
Peroxide value (meq O_2_/kg oil)	0.92 ± 0.02 b	1.25 ± 0.01 a	1.26 ± 0.01 a	0.10 ± 0.04 b
Acid value (mg KOH/g oil)	2.20 ± 0.02 a	2.67 ± 0.01 b	2.20 ± 0.02 b	0.55 ± 0.01 c
*p*‐Anisidine value	4.60 ± 0.04 b	3.7 ± 0.10 c	5.30 ± 0.1 a	2.60 ± 0.2 d
TOTOX value	6.44 ± 0.03 b	6.20 ± 0.10 b	7.80 ± 0.1 a	2.80 ± 0.17 c
Oxidative stability index (OSI)	4.60 ± 0.12 a	3.70 ± 0.06 b	4.80 ± 0.11 a	3.40 ± 0.08 c
Total tocopherol content (mg α‐tocopherol/kg oil)	2053 ± 17 a	1689 ± 46 b	1439 ± 65 c	1053 ± 32 d
Total phenolic content (mg/kg oil)	130 ± 0.1 d	181 ± 1 c	199.3 ± 0.1 b	79.0 ± 0.2 a
DPPH radical scavenging power (%)	70.7 ± 0.6 b	35.9 ± 0.2 c	78.8 ± 3.1 a	32.0 ± 0.9 d
FRAP assay (mmol Fe^+2^/L oil)	746 ± 7 b	746 ± 1 a	748 ± 6 a	425 ± 3 c

*Note*: Each test was performed in three replications. Mean ± SD within a row with the same lowercase letters are not significantly different at α = 0.05.

Cox value is an index calculated based on the composition of fatty acids, reflecting the oxidative stability of edible oils. The lower the Cox value, the better the oxidative stability of the oil (Farhoosh et al., 2008). The oxidative stability also depends on other important factors, such as the total amount and type of antioxidants in an oil. The Cox values of the three pomegranate seed oils exceeded that of the oil from the sesame cultivar Dezful (Table 1). Therefore, based on that index's value, the “Dezful” oil comparatively has the best oxidative stability. Among the seed oils of three pomegranate cultivars, the best Cox value was in the cultivar Torsh Sabz. However, there was little difference between these three oils and no significant difference between the Shirin Khafr and Rabab cultivars. In previous studies, the levels of this index in oils from olive, sesame, *Pistacia atlantica* L., and *Pistacia vera* L. cv *Ohadi* kernel were reported to be 2.75, 6.24, 3.99, and 4.41, respectively (Farhoosh et al., 2008; Farhoosh & Tavakoli, 2008; Tavakoli et al., 2013); as such these were comparatively much smaller compared to the Cox value of the pomegranate seed oils accrued in this study.

The iodine value of the pomegranate seed oils, an index used to determine the degree of unsaturation in fats, highly exceeded that of the oil from sesame “Dezful” (Table 1). The difference in the iodine value of the oils may be attributed to their fatty acid structure. In a previous study of six pomegranate seed oils (Juhaimi et al., 2017), the iodine value content ranged between 214 (g *I* ^2^/100 g oil) and 218. In a study on the chemical structure of seed oil of five pomegranate cultivars cultivated in Iran, their iodine value was reported between 225 and 232 (Habibnia et al., 2012), which is close to the results of the present study. The iodine value of common edible oils has a wide range, with the most saturated coconut oil having an iodine value ranging from 4 to 9 (Marina et al., 2009). On the contrary, fish oil is the most unsaturated, with an iodine value between 142 and 176 (Oswell et al., 2020).

Based solely on the results of the three tests (fatty acid composition, Cox value, and iodine value), pomegranate seed oils are expected to have low oxidative stability due to high unsaturation. However, other factors, such as the amount of antioxidant compounds and the antioxidant power of these compounds, are also effective in oxidative stability.

#### 3.1.2 Unsaponifiable matters

Unsaponifiable matters of edible oils include tocopherols, polyphenols, sterols, hydrocarbons, and carotenoids. These compounds have antioxidative properties and high nutritional value and indicate the quality of edible oils (Kmiecik et al., 2022; Kulczyński et al., 2017; Różańska et al., 2019). The amount of unsaponifiable matter of the seed oils from three pomegranate cultivars and the oil from “Dezful” were comparable (Table 1). In a study conducted on seed oils from various pomegranate cultivars in the Saveh region of Iran, these compounds ranged between 1.47 and 1.83% (Habibnia et al., 2012), lower than the results of the present study. Differences in the characteristics of pomegranate cultivars and the cultivation area's climate can cause these differences: The amount of unsaponifiable matter of six cultivars grown in Turkey ranged between 1.17 and 2.07% (Juhaimi et al., 2017). The values of this index in cottonseed, virgin olive, canola, soybean, sesame, and corn oils are 0.7, 0.5, 0 to 1.5, 2, 1.6, 0.9 to 2.3, and 2.8%, respectively (Kochhar et al., 2020).

### 3.2 Initial qualitative parameters

To evaluate the initial quality of edible oils, the rate of hydrolysis of triglycerides, their primary oxidation, and their secondary oxidation must be determined simultaneously. These are informed by the tests of acid value, peroxide value, and *p*‐anisidine value, respectively. The results of the mentioned tests are given in Table 1. The lowest acid value was observed in “Dezful” oil (0.55), highly exceeded by all three pomegranate seed oils, with no significant difference between “Rabab” and “Torsh Sabz” oils. Moreover, the peroxide values in the three pomegranate seed oils and the “Dezful” oil were generally comparable. The amount of oxidation in the pomegranate seed oils was in the desirable range. The results of the *p*‐anisidine test showed that the “Dezful” oil (2.6) had the lowest secondary oxidation, exceeded by the three pomegranate seed oils. The initial quality of the four oils analyzed in this study showed that the “Dezful” oil had the best initial quality, followed by “Shirin Khafr,” “Torsh Sabz,” and “Rabab” oils. The differences in the initial quality of edible oils can be attributed to improper harvesting, handling, storage, and processing conditions (List et al., 2020).

### 3.3 Rancimat test

The Rancimat test is the most common accelerated method for assessing edible oils and fats' oxidative stability and antioxidative activity. The oxidative stability index (OSI) values of all four analyzed seed oils were generally comparable (Table 1), with no significant differences between Shirin Khafr and Rabab oils. In a previous study of seed oils from five cultivars of Iranian pomegranates, their OSI values determined using the Rancimat test were determined between 0.77 to 1.02 h (Habibnia et al., 2012), which was very low compared to the results of the present study. The OSI level of commercial pomegranate oils prepared from fruits grown in Israel and Turkey is between 0.1 and 0.22 h (Costa et al., 2019). The phenolic compounds of these oils were reported to be between 2.8 and 168 mg/kg. The difference between the results of the present research and previous studies may be attributed to storage conditions, the amount and type of the antioxidant compounds, as well as the unsaturation levels of the edible oils.

### 3.4 Thermal processing

#### 3.4.1 Oxidative stability changes

**FIGURE 1:**
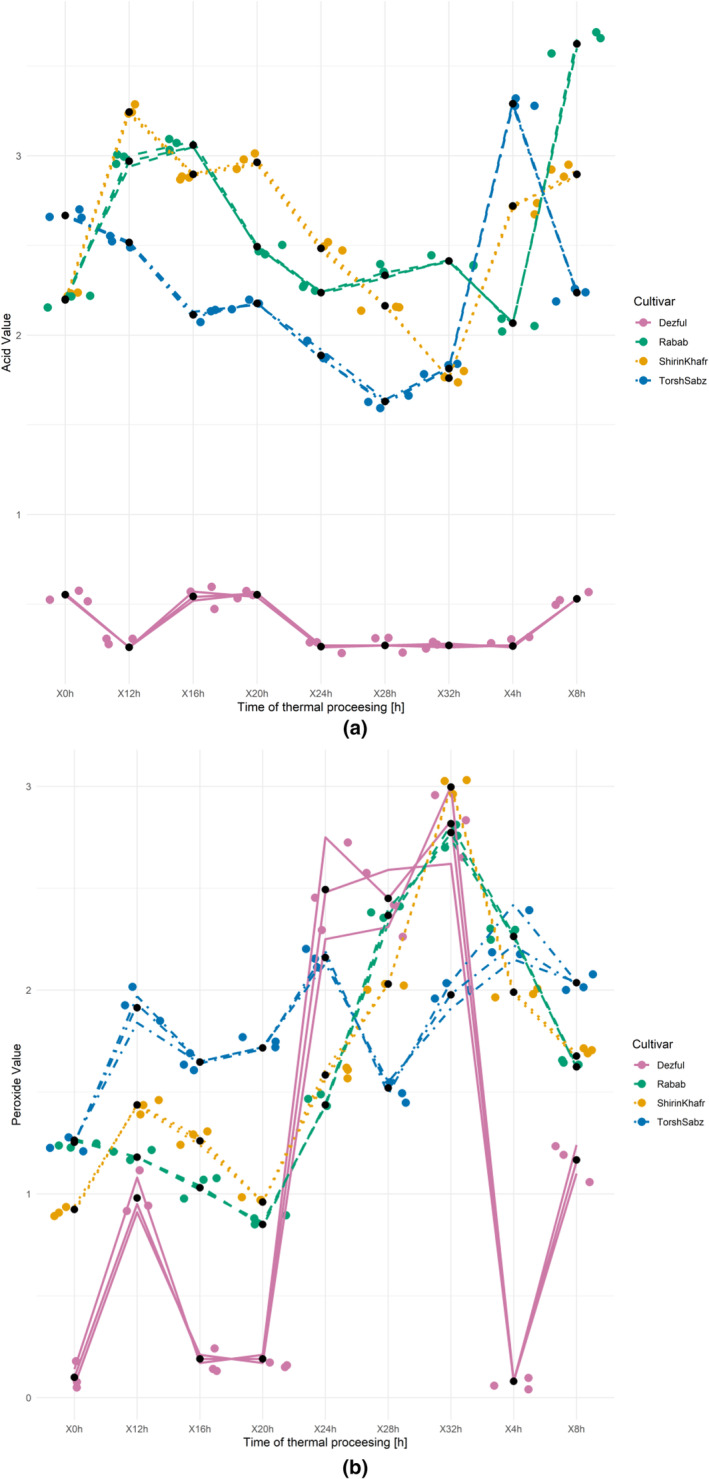
Changes in the acid value (a) and the peroxide value (b) of seed oils from three cultivars of pomegranate (Shirin Khafr, Torsh Sabz, and Rabab) and one cultivar of sesame (Dezful) during the thermal processing at 170°C for 32 h.

The acid value reflects the rate of hydrolysis of triglycerides to free fatty acids. As the fatty acids are released from the triglycerides, the oxidation rate in edible oils increases exponentially. For this reason, the storage conditions of edible oils should prevent the hydrolysis process. The presence of moisture or the lipase enzyme is the most common reason for the hydrolysis of triglycerides (Oswell et al., 2020). Changes in the acid value of different oils in this study during the thermal processing for 32 h are shown in Figure 1a. There were no obvious trends in the changes in the acid value, especially for the pomegranate seed oils. Usually, the acid value is expected to increase during thermal processing. However, in the present study, a decrease in this index was also observed in addition to the cross‐sectional increase in the acid value. After 32 h of thermal processing, 20, 32, and 52% decreases were observed compared to the initial acid values in the Shirin Khafr, Torsh Sabz, and Dezful oils, respectively. Only the “Rabab” oil showed a 10% increase in acid value at 32 h compared to the starting value. Among the oils analyzed in the present study, the most regular trend was observed in the “Dezful” oil. The acid value of crude Kolkhoung (*Pistacia Shinjuk*u) hull oil was reduced during the thermal processing due to the evaporation of free fatty acids or the binding of these acids to other compounds during polymerization (Tavakoli et al., 2013). Due to the lack of a refining process and various compounds in crude oils, the free fatty acids can bind with other compounds. In a study on seed oils from pomegranate cultivars (Juhaimi et al., 2017), their acid values ranged between 4.21 and 7.51, much higher than the starting or maximal acid values of the pomegranate oils in this study. In some other studies that have used antioxidative oils with very high oxidative stability, in contrast to the present study, clear ascending trends were observed in the acid value during the thermal processing. In two different studies, Baneh (*Pistacia atlantica*) kernel oil and its unsaponifiable matter were used to improve the thermal stability of the canola oil. Changes in acid values during the 48 h of thermal processing were ascending (Sharayei et al., 2011a, 2011b). Similarly, the acid values of virgin coconut oil during the frying process also increased (Srivastava & Semwal, 2015).

The known mechanism of oxidation of edible fats and oils includes the initiation, propagation, and termination stages; during this initial oxidation process, the content of hydroperoxides increases exponentially under normal storage conditions. Subsequently, the hydroperoxides break down due to inherent instability, and their content decreases over time as the carbonyl compounds are formed. This stage is called secondary oxidation (Farhoosh et al., 2008). Changes in the peroxide value that measures the secondary oxidation of the oils during 32 h of thermal processing at 170°C are presented in Figure 1b. There were no clear trends in the peroxide value changes in the tested oil samples.

The increase in peroxide value of seed oils from “Shirin Khafr,” “Torsh Sabz,” “Rabab,” and “Dezful” after 32 h of thermal processing compared to initial values was 224, 58, 120, and 2745%, respectively. In a similar study aiming at assessing the effect of heat treatment at 120°C on *P. khinjuk* kernel and hull oils, the rate of increase in peroxide value after 32 h of thermal processing was 1212 and 1361%, compared to the starting values, respectively (Asnaashari et al., 2015), which was very high compared to the present study. In other related studies, similar results were obtained (Aşkın & Kaya, 2020; Gao et al., 2020; Kmiecik et al., 2020; Matthäus, 2006).

**FIGURE 2:**
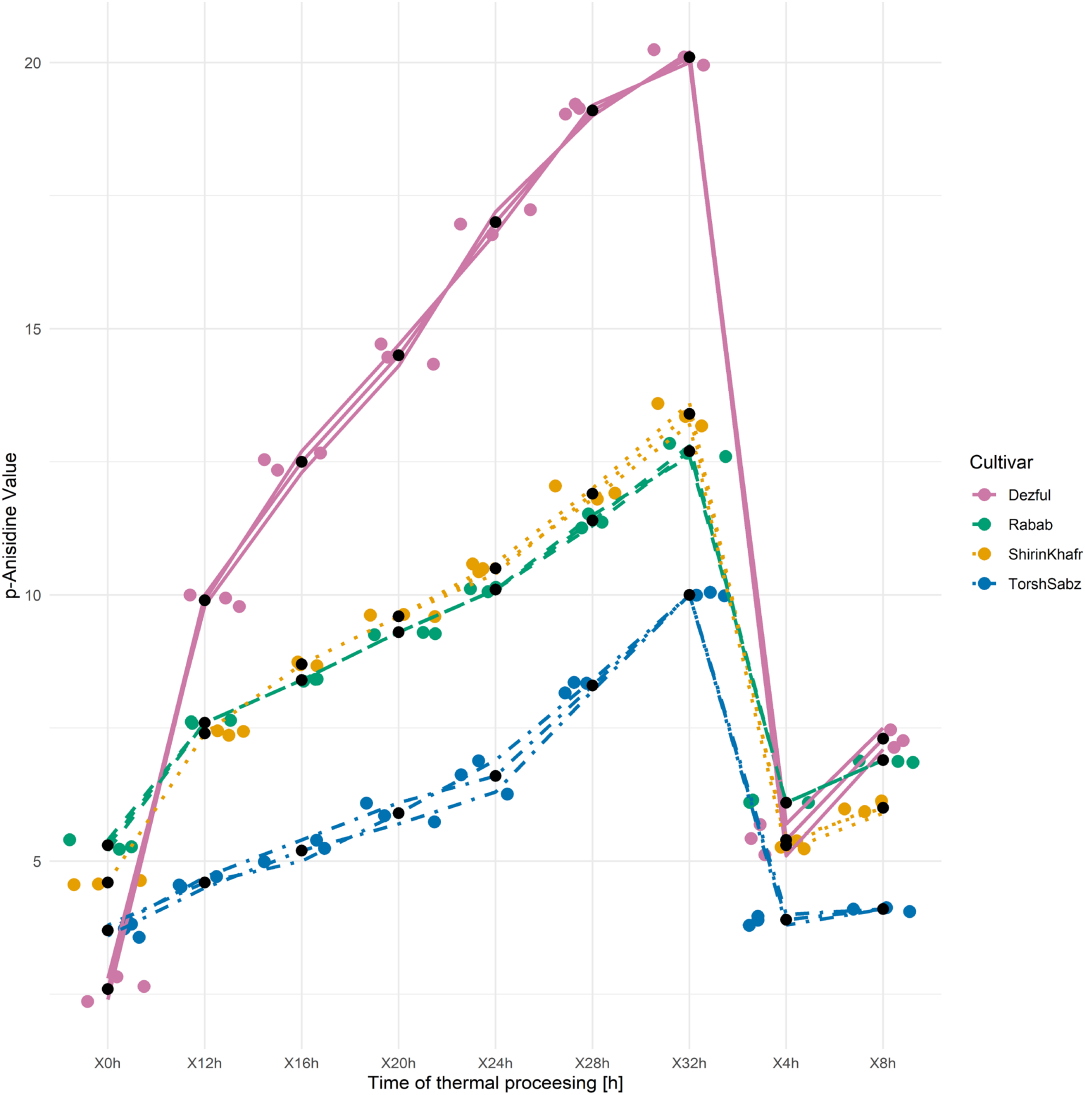
Changes in the *p*‐anisidine value of seed oils from three cultivars of pomegranate (Shirin Khafr, Torsh Sabz, and Rabab) and one cultivar of sesame (Dezful) during the thermal processing at 170°C for 32 h.

Hydroperoxides are formed intermittently at temperatures above 100°C and then decompose rapidly. Carbonyl compounds are formed at such temperatures by the decomposition of hydroperoxides and are indicators of secondary oxidation, as determined by the *p*‐anisidine value. Changes in the *p*‐anisidine value of the analyzed oils during the thermal processing are depicted in Figure 2. The *p*‐anisidine values increased linearly throughout the thermal processing. Among the analyzed seed oils, the “Dezful” oil increased with the steepest slope of 2.25 during the thermal processing, and “Torsh Sabz” with the mildest slope of 0.74, respectively. The increase in p‐anisidine value of seed oils from “Shirin Khafr,” “Torsh Sabz,” “Rabab,” and “Dezful” was 192, 169, 141, and 678%, respectively. Comparably, the analyses of oxidative stability of *P. khinjuk* kernel and hull oils after 32 h of thermal processing at 170°C showed that the rates of increase in the *p*‐anisidine value compared to starting values were 607 and 603% (Asnaashari et al., 2015). Several studies have shown that *P. khinjuk* kernel and hull oils have exceptional oxidative stability and antioxidative activity and compete with the powerful antioxidant TBHQ (Tavakoli et al., 2013, 2018; Tavakoli & Sorbi, 2017). The superiority of the pomegranate seed oils analyzed in this study using the test of *p*‐anisidine value (% increase) over the *P. khinjuk* kernel and hull oils indicates their very good oxidative stability. In a study that analyzed the effects of pomegranate seed blanching at 95°C for 3 min on the quality of the extracted oils, the *p*‐anisidine value of the pomegranate seed oil during this process increased from 1.97 to 2.71 (Kaseke et al., 2021).

**FIGURE 3:**
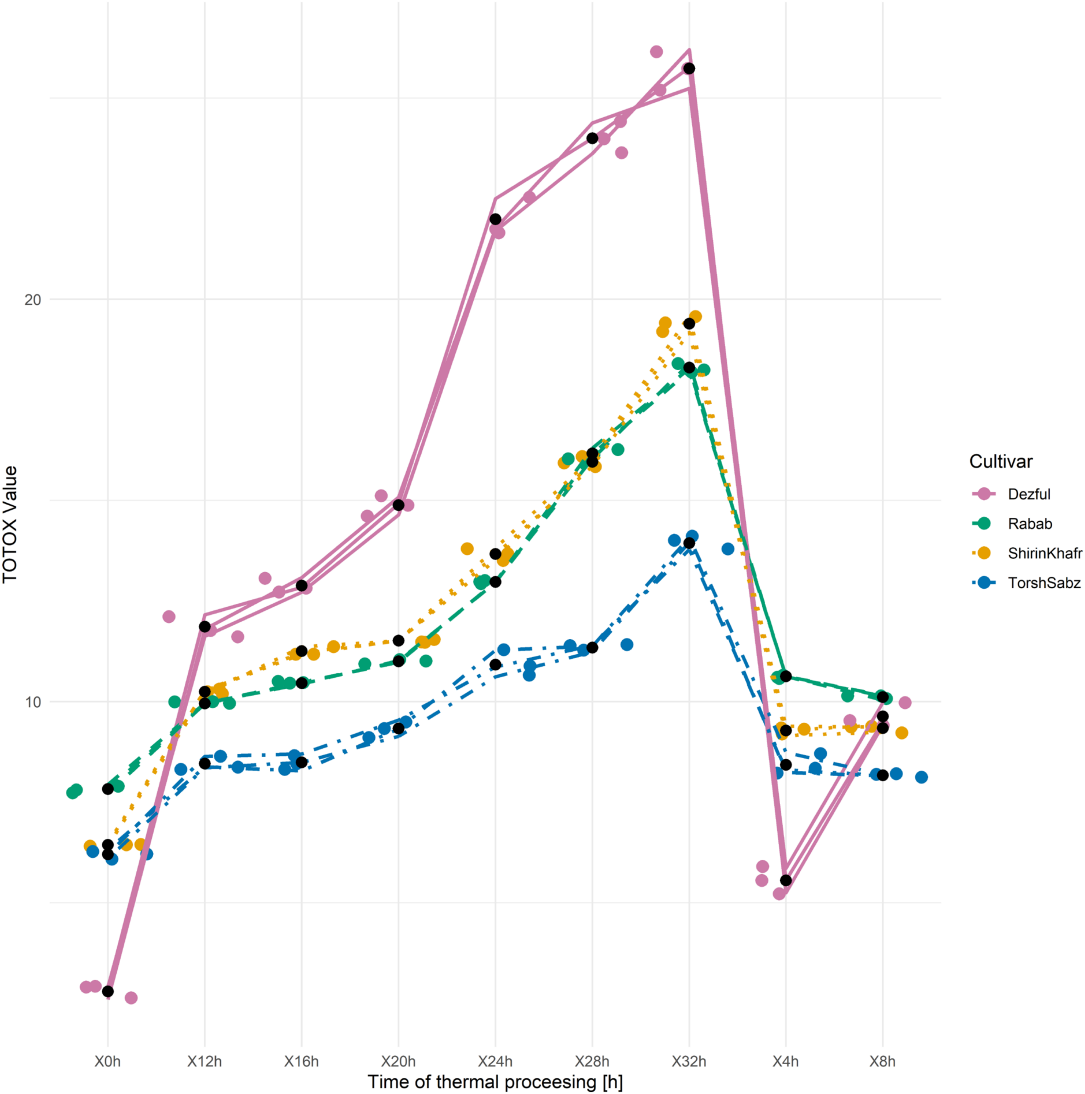
Changes in the TOTOX value of seed oil from three cultivars of pomegranate (Shirin Khafr, Torsh Sabz, and Rabab) and one cultivar of sesame (Dezful) during the thermal processing at 170°C for 32 h.

To assess the status of edible oils, it is necessary to perform several analyses. TOTOX value measures the total oxidation that includes the products of primary and secondary oxidations. The peroxide test measures the intensity of the primary oxidation and does not inform about any changes in compounds resulting from the secondary oxidation. Likewise, the *p*‐anisidine value, as an indicator of secondary oxidation, is useful only in frying edible oils because, under high temperatures, the hydroperoxides decompose and their amount decreases. For this reason, using the TOTOX value to obtain accurate information about edible oils is necessary. This index thus represents total oxidation equal to the sum of the twice peroxide value and the *p*‐anisidine value (Wai et al., 2009). The TOTOX value changes of the analyzed seed oils during the thermal processing are shown in Figure 3. The lowest increases of this test results were seen in the pomegranate seed oils (124–201%), in contrast to “Dezful” (820%). The TOTOX of pomegranate seed oils generally showed good oxidative stability; among them, “Torsh Sabz” oil had the most desirable value. One of the reasons for the superiority of this oil can be related to its lower Cox value compared to the other two pomegranate oils. Comparably, the “Dezful” oil, with its lowest Cox value, showed the worst property regarding oxidation resistance. Other factors, such as the levels of antioxidants and the type of antioxidants, affect the oxidative stability of edible oils (Habibnia et al., 2012; Mildner‐Szkudlarz et al., 2019).

#### 3.4.2 Changes in the content of antioxidants (tocopherol and phenolic compounds)

Tocopherols are the major hydrophobic antioxidant compounds. Total tocopherol levels in the seed oils from “Shirin Khafr,” “Torsh Sabz,” and “Rabab” substantially exceeded that in “Dezful” (Table 1). Contents of tocopherols were much higher in the pomegranate seed oils than in the conventional edible oils. The amount of tocopherol in walnut, cottonseed, canola, olive, palm, peanut, soybean, and sunflower oils is 830, 900 to 1500, 690 to 695, 30 to 300, 360 to 560, 330 to 480, 900 to 1400, and 630 to 700 mg/kg, respectively (Oswell et al., 2020). The tocopherols reported for seed oils from five pomegranate cultivars cultivated in the Saveh region of Iran ranged from 544 to 1135 mg/kg (Habibnia et al., 2012). In a study that investigated the chemical structure of seed oil of six pomegranate cultivars in Turkey, total tocopherol compound content ranged between 3427 and 4631 mg/kg; of these, α‐tocopherol was the predominant species in all oils (Juhaimi et al., 2017). In the seed oil of six pomegranate cultivars grown in Tunisia, the tocopherol content ranged between 2780 and 3180 mg/kg (Elfalleh et al., 2011). Comparatively, in the seed oil of nine pomegranate cultivars, their tocopherol content ranged between 1745 and 6273 mg/kg (Fernandes et al., 2015).

**FIGURE 4:**
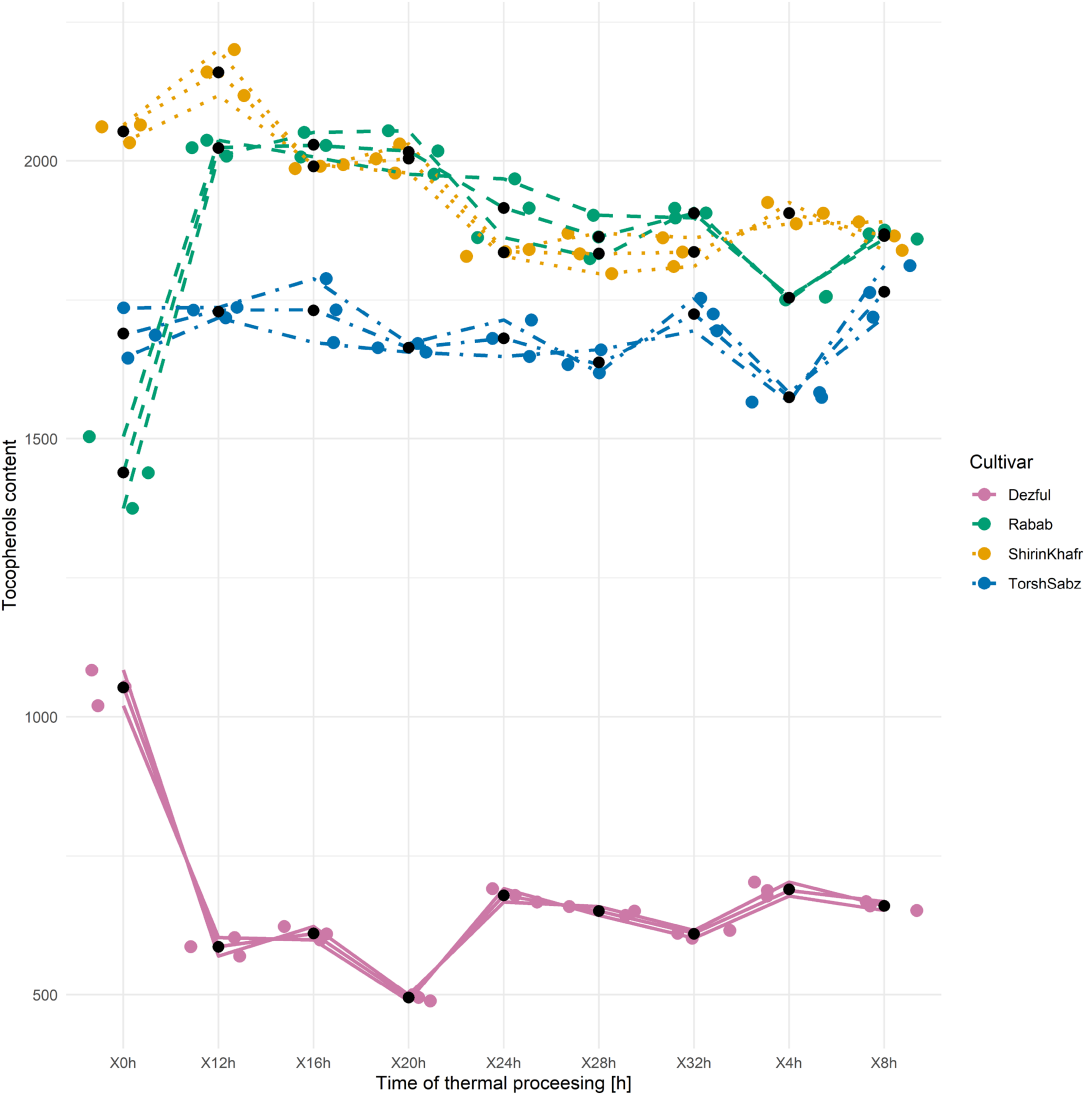
Changes in the total tocopherol compounds of seed oil from three cultivars of pomegranate (Shirin Khafr, Torsh Sabz, and Rabab) and one cultivar of sesame (Dezful) during the thermal processing at 170°C for 32 h.

The 32‐h‐long thermal processing varied in its effects on the total tocopherol levels (Figure 4). The levels of tocopherols in “Rabab” and “Torsh Sabz” oils increased by 32.5 and 2.1%, respectively, at the end of the thermal processing compared to their starting values. In contrast, tocopherols decreased by 10.5 and 42.1% in the seed oils from “Shirin Khafr” and “Dezful,” respectively. It is usually expected that the tocopherol levels would decrease during the thermal process due to thermal decomposition, and this has been reported in various studies (Sharayei et al., 2011a, 2011b; Tavakoli & Sorbi, 2017). Nevertheless, the present study observed increased pomegranate seed oil tocopherols during thermal processing. In some previous studies on edible oils, increases in tocopherol levels have been reported during various processes, such as heat or refining. In research, Veronezi and Jorge reported that applying the thermal process at 180°C on the oil‐containing soybean, papaya, and melon oils (60:20:20) increased the amount of tocopherols compared to the zero moments. The amount of tocopherols at 0, 10, and 20 h of the thermal process was 383, 354, and 400 mg/kg, respectively. Among tocopherol compounds, the highest increases were observed in α‐tocopherol and γ‐tocopherol, respectively (Veronezi & Jorge, 2018).

This finding may be related to the phenomenon of tocopherol regeneration. Tocopherols are not always free and may form a matrix with themselves or other compounds. Breaking these bonds releases tocopherols, and their increase can be observed. Some of these compounds were present in the oil and could not be traced when connected to other compounds, such as sterols. Tocopherols were released and regenerated during the thermal processing, increasing their content at several time points. It is also possible that tocoquinones, which are the products from the oxidation of tocopherols, can be converted back into tocopherols in the presence of reducing compounds, and the increase in these compounds can be observed in some hours of the thermal process (Jurasova et al., 2023; Kaşıkçı & Bağdatlıoğlu, 2022; Nekouei & Rezaei, 2020).

Phenolic compounds are natural antioxidants and represent important factors that determine the quality of edible oils. A direct relationship was established experimentally between the levels of phenolic compounds and the oxidative stability and organoleptic properties of edible oils. Phenolics also have biological roles in the human body by increasing the antioxidant defense capacity and thereby preventing diseases caused by the formation of excessive free radicals (Farhoosh et al., 2008; Tavakoli et al., 2016). The initial phenolic levels of seed oils from “Shirin Khafr,” “Torsh Sabz,” and “Rabab” varied significantly and exceeded that of “Dezful” (Table 1). Levels of phenolics in these oils reached 6.4, 10.7, 13.9, and 7.5% of their tocopherol levels, respectively. Therefore, phenolics amount to a small part of the antioxidant capacity of these oils. Several studies have reported on the levels of phenolics in pomegranate seed oils. Phenolics of seed oils of six pomegranate cultivars grown in Georgia ranged from 849 to 911 mg/kg (Grossmann et al., 2010). In other studies, phenolics of pomegranate seed oils grown in South Africa (Kaseke et al., 2021) and Serbia (Drinić et al., 2020) were reported at 1200 and 160 mg/kg, respectively. In the seed oils of three pomegranate cultivars cultivated in Italy, phenolics were assessed at between 4100 and 6850 mg/kg (Passafiume et al., 2019). In the pomegranate seed oils from various regions of Turkey and Israel, phenolic levels ranged between 2.8 and 168 mg/kg (Sharayei et al., 2011b). There is a wide range of phenolic levels, which can be attributed to genetic and environmental factors.

**FIGURE 5:**
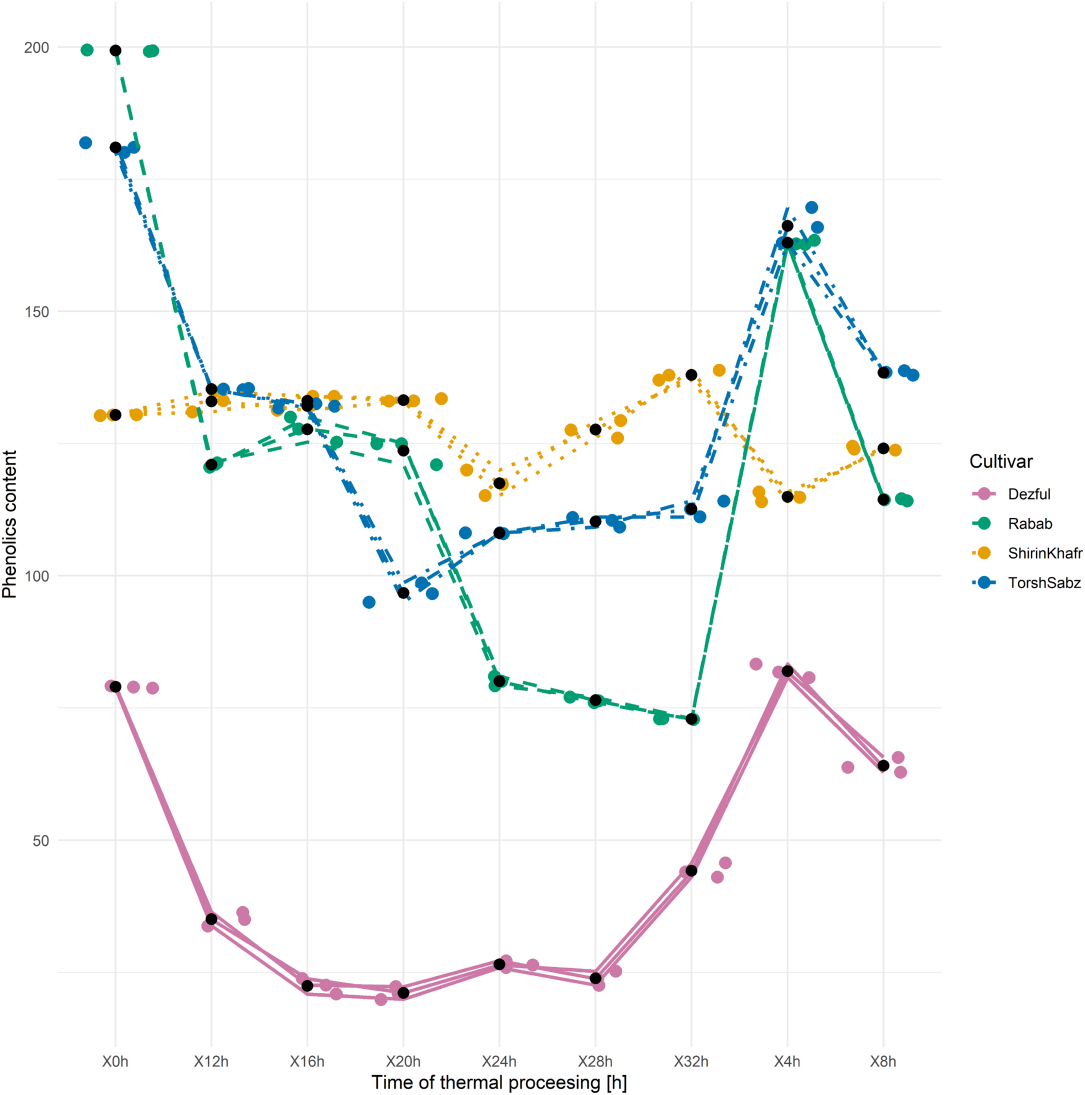
Changes in the total phenolic compounds of seed oil from three cultivars of pomegranate (Shirin Khafr, Torsh Sabz, and Rabab) and one cultivar of sesame (Dezful) during the thermal processing at 120°C for 32 h.

Changes in the phenolic content of the analyzed oils during 32 h of thermal processing are depicted in Figure 5. At the end of the thermal processing, compared to their starting values, the content of phenolics in “Torsh Sabz,” “Rabab,” and “Dezful” oils decreased by 37.8, 63.4, and 44.1%, respectively, but in the case of “Shirin Khafr” oil, it increased by 5.8%. Slight increases in the phenolic levels were also observed at several time points of the thermal processing. The results of the thermal processing of olive oil at 180°C showed that the phenolics of virgin and refined oil from olive (Bladi cultivar) increased by 39% (after 1 h of the thermal process) and 42% (after 2 h of the thermal process), respectively (Mirrezaie Roodaki et al., 2016). Similar was reported for the thermal processing of oils from soybean, papaya, and melon at 180°C for 20 h (Veronezi & Jorge, 2018); the combined post‐processing formulations of these oils had very sharp phenolics increases compared to the initial levels, attributed to phenolics formed during the thermal decomposition of tocopherols. A study of the oxidative stability of pomegranate seed crude oil for 12 days at 65°C indicated a progressing decrease in the phenolics content (Drinić et al., 2020). At the end of that storage period, phenolics decreased by 60%, likely due to their antioxidant role in oxidative reactions. Differences in the phenolics species, temperature, and storage time can underlie the discordant results (Drinić et al., 2020) and the present study. Comparably, the levels of phenolics in the crude olive oil were reduced by 53% during 1 h of heating processing at 180°C (Battino et al., 2002). The difference between that study and the present one can be attributed to the huge difference in thermal processing time. With 1 h of thermal processing, the reactions that lead to increased phenolics are likely impossible. To that point, the phenolic content of hazelnut oil increased and decreased during the frying process (Karakaya & Şimşek, 2011).

Changes in tocopherol and polyphenolic compounds during the thermal process are the reason for the higher oxidative stability of pomegranate seed oils compared to sesame oil. These compounds were much higher in unheated pomegranate kernel oils than in sesame oil. Also, during the thermal process, a lower reduction in tocopherol and polyphenolic compounds was observed compared to sesame oil. The mentioned items are directly related to the oxidative stability of edible oils.

#### 3.4.3 Antioxidative activity changes

Inhibition of free radicals is one of the most common mechanisms determining the activity of antioxidants and antioxidative capacity in oils. The initial free radical scavenging power in seed oils from “Shirin Khafr,” “Torsh Sabz,” and “Rabab” exceeded that of the “Dezful” oil (Table 1), and for all, analyzed oils did not correlate with their initial levels of antioxidant compounds including phenolics and tocopherols. The free radical scavenging of seed oils from six pomegranate cultivars grown in Turkey ranged between 17.5 and 22.9% (Juhaimi et al., 2017). Another study reported pomegranate seed oils' free radical scavenging power at 22% (Drinić et al., 2020).

**FIGURE 6:**
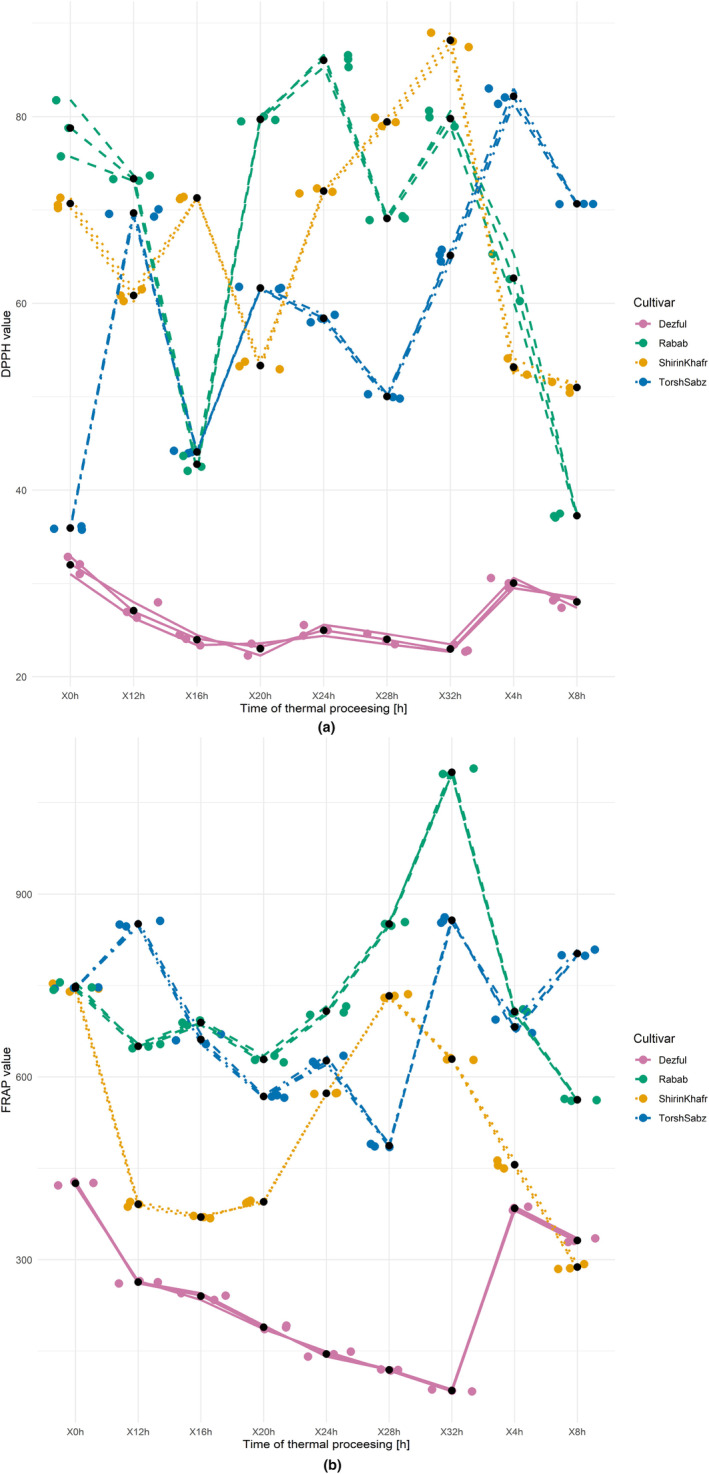
Changes in the DPPH radical scavenging power (a) and FRAP assay (b) of seed oil from three cultivars of pomegranate (Shirin Khafr, Torsh Sabz, and Rabab) and one cultivar of sesame (Dezful) during the thermal processing at 170°C for 32 h.

During the thermal processing of pomegranate seed oils (Figure 6a), the free radical scavenging power changes were irregular. However, the seed oil from sesame “Dezful” showed a linear decreasing trend. These results can be attributed to changes in the antioxidant compounds during the thermal processing, mainly tocopherols and phenolics. The irregular antioxidant levels in the pomegranate seed oils discussed above may have affected their free radical scavenging power. As for the “Dezful” oil, the conditions for changes in the antioxidant levels were more regular over time and generally decreased, correlating with the free radical scavenging power during the thermal processing. At the end of that processing, the free radical scavenging power of “Shirin Khafr,” “Torsh Sabz,” and “Rabab” oils increased by 24.7, 81.2, and 1.4% compared to their respective starting values, but for the “Dezful” oil, it decreased by 32.4%. The oxidative stability of pomegranate seed oils during 12 days of thermal processing at 65°C was investigated in previous studies. Levels of the free radical scavenging power of DPPH were decreasing so that after the storage period, the amount of free radical scavenging power decreased from the initial value of 22 to 8%, consistent with the noted changes in phenolics levels (Drinić et al., 2020). However, for oils from papaya and melon that served as additives to the soybean oil during 20 h of thermal processing at 180°C, both the added oils and their combination with the soybean oil showed an increase in free radical scavenging power. Then, a decrease was observed in some samples, consistent with the present research results.

FRAP test is a simple, repeatable, and cost‐effective way to determine the reducing capacity of Fe^+3^ to Fe^+2^. The initial values of this test in seed oils from “Shirin Khafr,” “Torsh Sabz,” and “Rabab” greatly exceeded the FRAP of the “Dezful” oil (Table 1). The FRAP oil test of three other pomegranate cultivars reached 6090, 3000, and 1950 mmol/L, respectively (Kaseke et al., 2020). These values decreased 82% after microwave treatment but showed no significant difference in the “Acco” seed oil. Changes in FRAP during 32 h of thermal processing (Figure 6b) showed irregular patterns in the pomegranate seed oils. However, in the case of oil from sesame “Dezful,” a clear linear decreasing trend was noted. By examining the reducing capacity of oils at the end of the thermal processing compared to their initial values, increases of 15 and 47% were observed in “Torsh Sabz” and “Rabab” oils and decreases of 16 and 80% in “Shirin Khafr” and “Dezful” oils, respectively.

The observed increases in FRAP at several time points during the thermal processing of the pomegranate seed oils were likely due to changes in the levels of antioxidant compounds. In the test of soybean oil after 10 and 20 h of heat treatment at 180°C (Veronezi & Jorge, 2018), FRAP increased by 65 and 50%, respectively, compared to the starting value. The reason was attributed to the changes in the content of antioxidant compounds. The levels of phenolics and tocopherols in soybean oil increased by 202% and decreased by 74%, respectively, compared to the initial values (Veronezi & Jorge, 2018).

## 4 CONCLUSIONS

Based on the fatty acid composition of pomegranate seed oil, it is thought that it does not have good oxidation stability at high temperatures. However, the present research results showed that the pomegranate seed oils studied in this project had strong oxidative stability at high temperatures. One of the reasons for these results was the presence of a very high amount of antioxidant compounds, especially tocopherols, in the pomegranate seed oils. Also, changing the tocopherol compounds during the thermal process was quite effective. Observing the phenomenon of regeneration of tocopherols due to heating was one of the interesting findings of this research. This finding means that the thermal process positively affected pomegranate seed oils. Based on the accrued results, the pomegranate seed oil can be used as an additive to other oils to improve their oxidation stability in intense thermal processing, to be confirmed by future research.

## AUTHOR CONTRIBUTIONS

**Javad Tavakoli:** Formal analysis (equal); funding acquisition (equal); methodology (equal); project administration (equal); validation (equal); visualization (equal); writing – original draft (equal); writing – review and editing (equal). **Afsaneh Ghorbani:** Data curation (equal); formal analysis (equal); funding acquisition (equal); investigation (equal); project administration (equal); resources (equal); software (equal); writing – review and editing (equal). **Abdollah Hematian Sourki:** Conceptualization (equal); data curation (equal); formal analysis (equal); funding acquisition (equal); software (equal); visualization (equal); writing – review and editing (equal). **Askar Ghani:** Funding acquisition (equal); investigation (equal); software (equal); supervision (equal); visualization (equal); writing – original draft (equal). **Aniseh Zarei Jelyani:** Data curation (equal); formal analysis (equal); investigation (equal); supervision (equal); visualization (equal); writing – original draft (equal). **Przemysław Łukasz Kowalczewski:** Conceptualization (equal); formal analysis (equal); funding acquisition (equal); investigation (equal); software (equal); writing – original draft (equal). **Aynura Aliyeva:** Data curation (equal); formal analysis (equal); funding acquisition (equal); visualization (equal); writing – original draft (equal); writing – review and editing (equal). **Amin Mousavi Khaneghah:** Conceptualization (equal); investigation (equal); methodology (equal); supervision (equal); validation (equal); visualization (equal); writing – original draft (equal).

## CONFLICT OF INTEREST STATEMENT

The authors declare no conflict of interest.

## Data Availability

All data generated or analyzed during this study are included in this published article.
